# Ultraviolet-A radiation induces changes in cyclin G gene expression in mouse melanoma B16-F1 cells

**DOI:** 10.1186/1475-2867-7-7

**Published:** 2007-05-02

**Authors:** Riikka Pastila, Dariusz Leszczynski

**Affiliations:** 1 Non-ionizing Radiation Laboratory; STUK-Radiation and Nuclear Safety Authority, Helsinki, Finland; 2 Radiation Biology Laboratory; Department of Research and Environmental Surveillance, STUK-Radiation and Nuclear Safety Authority, Helsinki, Finland

## Abstract

**Background:**

We have previously shown that ultraviolet-A (UVA) radiation enhances metastatic lung colonization capacity of B16-F1 melanoma cells. The aim of this study was to examine changes in expression profile of genes in mouse melanoma B16-F1 cells exposed to UVA radiation.

**Results:**

B16-F1 melanoma cells were exposed to a single UVA radiation dose of 8 J/cm^2 ^and mRNA was isolated 4 h after the end of UVA exposure. Atlas™ Mouse Cancer 1.2 cDNA expression arrays were used for the large-scale screening to identify the genes involved in the regulation of carcinogenesis, tumor progression and metastasis. Physiologically relevant UVA dose induced differential expression in 9 genes in the UVA exposed melanoma cells as compared to the unexposed control cells. The expression of seven genes out of nine was upregulated (HSC70, HSP86, α-B-crystallin, GST mu2, Oxidative stress induced protein OSI, VEGF, cyclin G), whereas the expression of two genes was down-regulated (G-actin, non-muscle cofilin). The gene expression of cyclin G was mostly affected by UVA radiation, increasing by 4.85-folds 4 hour after exposure. The analysis of cyclin G protein expression revealed 1.36-fold increase at the 6 hour time point after UVA exposure. Cell cycle arrest in G2/M phase, which is known to be regulated by cyclin G, occurred at 4-h hour time-point, peaking 8 hours after the end of UVA irradiation, suggesting that cyclin G might play a role in the cell cycle arrest.

**Conclusion:**

Our results suggest that UVA radiation-induces changes in the expression of several genes. Some of these changes, e.g. in expression of cyclin G, possibly might affect cell physiology (cell cycle arrest).

## Background

Ultraviolet (UV) radiation is known to play a significant role in the development of skin cancer [1]. The major part of solar UV radiation which reaches the earth's surface consists primarily of UVA radiation (90–99%) with the minor component of UVB radiation (1–10%). Recently published studies have demonstrated that UVA radiation can modulate a variety of biochemical processes, some of which are involved in the malignant transformation of skin [2,3] and mutagenesis [4,5]. UVA is known to cause severe oxidative damage via reactive oxygen species (ROS) [6], which can damage lipids [7], DNA [8] and induce apoptosis [9,10]. UVA may also play a significant role in the induction and development non-melanoma and melanoma skin cancers [3,5,11-13].

We have previously shown *in vitro *that UVA increases adhesiveness of B16-F1 melanoma cells to endothelium and affects expression of the cell surface adhesion molecules [14]. We have also demonstrated *in vivo *that UVA irradiation enhanced the melanoma lung colonization potential in C57BL/6 mice [15]. In this study we have examined the effect of UVA radiation on the gene expression B16-F1 melanoma cells. Gene expression of 1176 tumor-related genes was analyzed using Atlas™ mouse cancer 1.2 cDNA array (Clonetech, USA). Obtained data shows that 9 genes were differentially expressed 4 hours after the exposure to UVA dose of 8 J/cm^2^. The upregulated genes are involved in the cell cycle regulation, stress response, and angiogenesis. The down-regulated genes are involved in building cytoskeleton and regulating cell motility. The most affected gene out of 1176 tumor-related genes was cyclin G. However, in spite of ~5-fold upregulation of cyclin G gene expression after UVA exposure, the protein expression levels were only moderately affected. However, this change was apparently of sufficient magnitude to induce G2/M cell cycle arrest.

## Results

Gene expression screening was performed 5 times (n = 5) using Atlas™ complementary (cDNA) mouse cancer 1.2 array that comprises of probes for 1176 most commonly altered genes in carcinogenesis. The microarray analysis has revealed that the physiologically relevant UVA dose induced differential expression of nine genes in UVA exposed melanoma cells (Table 1). Expression of seven genes was upregulated, involving in the stress response (HSC70, HSP86, α-B-crystallin), the oxidative stress (GST mu2, Oxidative stress induced protein), angiogenesis (VEGF), and the cell cycle regulation (cyclin G). Expression of two genes involved in cell motility was down-regulated (G-actin, non-muscle cofilin).

**Table 1:** Differentially expressed genes after UVA dose of 8 J/cm^2^

**Gene family **[Swiss prot Accession #]	**No. of arrays**^**a**^	**Control**^**b **^**± SD**	**UVA exposed**^**c **^**± SD**	**t-test**	**Ratio**^**d**^	**Function**
**Stress induced**						
Heat shock 86-kDa protein (HSP86) [P07901]	3 ↑	0,32 ± 0,23	1,04 ± 0,44	0,43	3,23	Belongs to HSP90 family; cytoplasmic molecular chaperone regulating the correct folding in the heat induced conformational changes.
Heat shock cognate 71-kDa (HSC70; HSP73), mouse homolog of human [P11142]	2 ↑ 1 ↓	0,15 ± 0,12	0,27 ± 0,18	0,39	1,81	Belongs to HSP70 family; molecular chaperone regulating the correct folding; found in melanoma cell lines [33].
Alpha crystallin B-subunit, mouse homolog of human [P02511]	3 ↑	0,07 ± 0,03	0,21± 0,07	0,05	3,26	Belongs to HSP20 family; found in mammalian transparent lens in eye induced by stress [34,35].
**Oxidative Stress**						
Oxidative stress-induced protein (OSI) [Q64337]	2 ↑ 1 ↓	0,16 ± 0,14	0,31± 0,15	0,27	1,92	Regulates metabolic responses to oxidative stress, were also induced [36]
glutathione S-transferase mu2 (GSTM2); [P15626]	3 ↑ 1 ↓	0,07 ± 0,04	0,14 ± 0,09	0,23	2,09	Belongs to the GST superfamily; prevents the toxic injuries; expressed in human melanoma cells [37] and in keratinocytes from human squamous cell carcinoma [38].
**Cell cycle control**						
cyclin G [O54779; P51945]	5 ↑	0,13 ± 0,04	0,65 ± 0,36	0,03	4,85	Contributes to G2/M arrest in response to DNA damage; a transcriptional target of the p53.
**Angiogenesis**						
Vascular endothelial growth factor (VEGF) [Q00731]	4 ↑	0,07 ± 0,03	0,22 ± 0,08	0,03	2,94	Promotes endothelial cell proliferation and migration in angiogenesis; permeabilizes the blood vessels; expression regulated by UVB in keratinocytes and fibroblasts [39,40].
**Skeleton & Motility protein**						
G-actin cytoplasmic [P02571; P14104]	3 ↓	1,02 ± 0,37	0,47 ± 0,18	0,10	0,47	Exists in all eukaryotic cells as a component of the cytoskeleton mediating cell motility. Polymerization leads to formation of filamentous F-actin.
non-muscle cofilin 1 (CFL1) [P18760]	3 ↓	0,40 ± 0,10	0,17 ± 0,01	0,05	0,41	Controls reversibly actin depolymerization from F-actin to G-actin causing an increase in the G-actin pool.

^***a ***^The number of arrays (out of five), in which differences in the gene expression were observed.; The arrows indicate the upregulation or downregulation of the gene. In the remaining arrays no difference was observed.

^***b ***^The average of the normalized gene intensity in control cells ± standard deviation;

^***c ***^The average of the normalized gene intensity in UVA exposed cells ± standard deviation.

^***d ***^Ratio of UVA exposed genes versus control genes.

**Figure 1:**
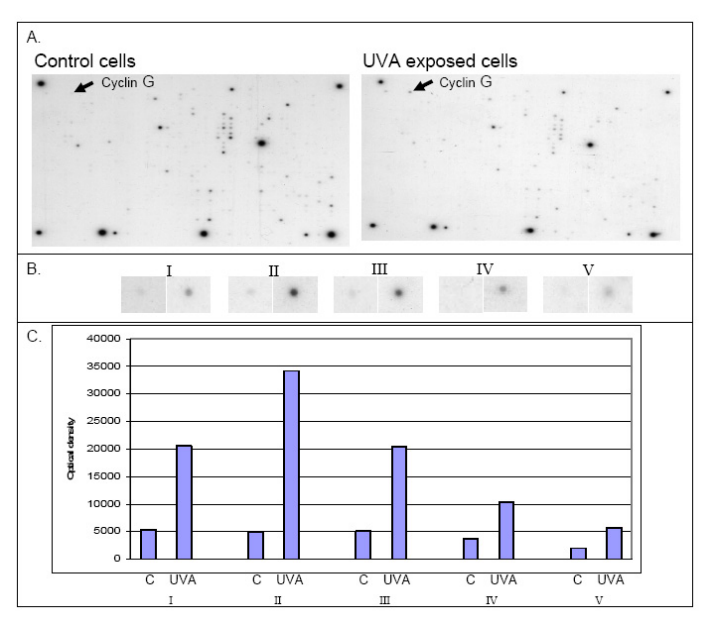
**cDNA array results of cyclin G**. **A**. The Representative image of Atlas™ Mouse Cancer 1.2 cDNA expression array hybridization film. Gene coding cyclin G, which was most prominently affected by UVA radiation, is indicated with an arrow at the corresponding position of two membranes. **B**. The upregulated cyclin G spots from cDNA arrays of five experiments at 4-hour time-point after the dose of 8 J/cm^2 ^of UVA. **C**. The optical densities of the cyclin G spots of five experiments.

**Figure 2:**
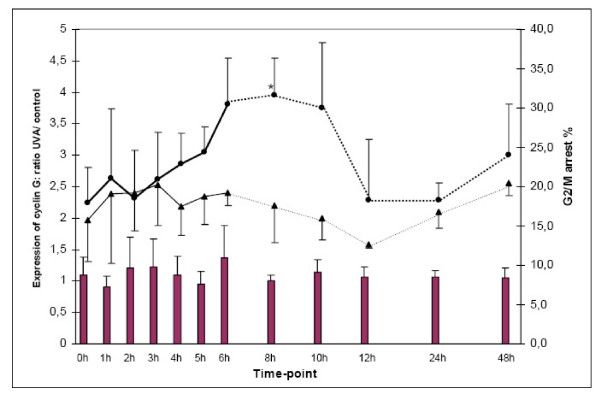
**The UVA effect on the protein expression of cyclin G and on the cell cycle arrest in B16-F1 cells**. The UVA effect on the protein expression of cyclin G in B16-F1 cell line is indicated by purple bars. The results are shown as the ratios, where the UVA treated value is divided by the control value. The cyclin G protein expression is moderately affected by UVA and there is a 1.36-fold increase in the cyclin G expression at 6-h time point. The UVA effect on the cell cycle arrest at the G2/M check point is indicated by black circles; non-exposed control cells are indicated by black triangles. The UVA exposure induces a significant cell cycle arrest, beginning at the 4-h time-point, peaking at the 8-h time-point in a statistically significant-manner (p < 0.05), and declining thereafter.

Cyclin G was examined further, since it was the most UVA-affected gene (Figure 1), being upregulated by 4.85-fold 4 h after UVA-exposure (Table 1). The protein expression of cyclin G in B16-F1 melanoma cells was examined immediately after the end of exposure and at different time-points thereafter (Figure 2). In spite of the statistically significant 4.85-fold upregulation in the gene expression, the cyclin G protein expression was only moderately affected by UVA. There was a 1.36-fold increase in the cyclin G protein expression at 6-h time point, being however statistically non-significant (Figure 2).

Since the cyclin G functions in the nucleus as the cell cycle regulator [16,17], the effect of UVA on the cell cycle was examined at the same time-points, where the protein analysis was performed. The UVA exposure induced time-dependent cell cycle arrest in G2/M phase of the cell cycle, beginning at the 4-h time-point, peaking at 8-h time-point (p < 0.05), and declining thereafter (Figure 2).

## Discussion

The aim of this study was to examine the UVA radiation induced changes in the gene expression in B16-F1 mouse melanoma cell line. B16-F1 melanoma cells were exposed to a single UVA radiation dose of 8 J/cm^2^, which roughly corresponds to the UVA dose received approximately within 1 hour on a sunny summer day in Finland.

Gene expression experiments showed that UVA affected expression of nine genes. In four of them (cyclin G, VEGF, α-crystallin and non-muscle cofilin) the change was statistically significant (p ≤ 0.05). The four hour time-point was selected in order to give melanoma cells time to respond to UVA radiation in transcriptional level. Furthermore, 4-h time-point was also used in our preliminary *in vitro *set-up, where we have observed alterations in the adhesive properties of the melanoma cells 4 hours after the end of irradiation [14].

The upregulation of heat-shock proteins observed in this study agrees with the previously shown induction of the overexpression of stress response proteins (Hsp70, Hsp86 and Hsp40) in human melanocytes in response to UVA [18]. The expression of vascular endothelial growth factor (VEGF) gene was upregulated in a statistically significant manner in this study, what is consistent with previously published observations, where UVA was shown to induce VEGF in dermal fibroblasts [19] and keratinocyte-derived cell lines [20]. This result suggests that UVA might affect melanoma tumor angiogenesis, via enhanced VEGF expression, and this hypothesis will be studied further in a separate project. Finally, UVA radiation exposure has caused decrease the G-actin and cofilin gene expression levels agreeing with earlier studies that have shown that UVA radiation decreases actin expression in human fibroblasts and keratinocytes [21], and in mouse and hamster fibroblasts [22].

The novel observation of this study was the UVA-induced 4.85-fold upregulation of the expression cyclin G gene. Cyclins are the regulatory subunits that control the progression and the check-points of the cell cycle. Cyclin G is a transcriptional target of the p53 tumor suppressor protein [23] and its growth inhibitory activity is linked to the ARF-mdm2-p53 and the retinoblastoma pathways [24,25]. Cyclin G is known to regulate the G2/M arrest, in response to DNA damage [16,17], for example after the UVC exposure [26]. In this study we have shown that cyclin G gene expression is regulated also by UVA radiation.

We have further examined, whether the upregulation of mRNA for cyclin G will lead to the increase in protein level in the UVA-exposed cells. Western blot analysis showed that the expression of cyclin G was affected by UVA radiation only moderately. The 1.36-fold increase in the cyclin G expression was seen at 6-h time point. We have also examined, whether UVA-induced changes in cyclin G expression (gene and protein) had functional effect on cell cycle [16,17]. Cell cycle analysis showed that UVA-exposure caused cell cycle arrest in G2/M phase beginning at the 4-h time-point and peaking 8 hours after the end of irradiation.

**Figure 3:**
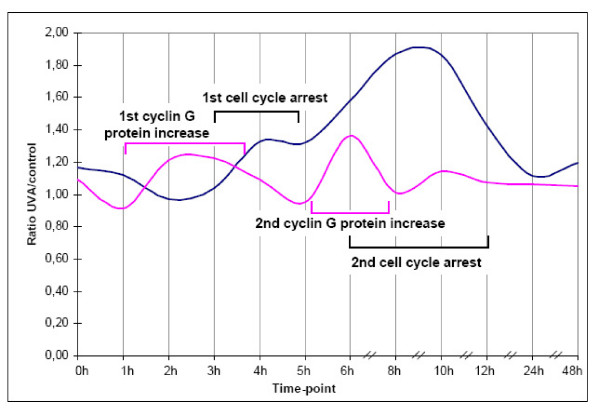
**Comparison of the timing of the cyclin G expression and cell cycle arrest in G2/M phase**. The results are expressed as the ratios. The moderate increase in the protein expression of cyclin G (pink line) at the 2-h and 3-h time-points precedes the G2/M cell cycle arrest (blue line), which starts 4 h after the end of the UVA irradiation. The second increase in the cyclin G protein expression observed at the 6-h time-point is followed by further increase in G2/M phase arrest.

Comparison of the timing of the cyclin G expression and cell cycle arrest in G2/M phase (Figure 3) shows that the moderate increase in the protein expression of cyclin G at 2-h and 3-h time-points preceded the cell cycle arrest observed starting at 4 h after the end of the UVA irradiation. Also the second increase in the cyclin G protein expression observed at 6-h time-point was followed by further increase in G2/M phase arrest. This suggests that UVA-induced changes in cyclin G expression might play some role, among other factors, in induction of cell cycle arrest. It is possible to hypothesize that cell cycle arrest might allow cells to repair UVA-induced DNA damage. This hypothesis, however, requires further study.

## Conclusion

In conclusion, our data suggest that UVA is capable to alter the expression of several genes in B16-F1 mouse melanoma cell line. Some of these changes, e.g. expression of cyclin G, might affect cell physiology (cell cycle). The biological and physiological significance of these *in vitro *observed changes needs to be corroborated by the *in vivo *study in human volunteers before any health-related conclusions and hypotheses can be drawn.

## Methods

### Cell culture

Mouse melanoma B16-F1 cell line [27] was obtained from National Cancer Institute, Frederick Cancer Research and Development Center (Frederick, MD). B16-F1 melanoma cell line was cultured in RPMI 1640 and was supplemented with 10% heat-inactivated FBS, penicillin-streptomycin (100 IU/ml-100 μg/ml), and L-glutamine (4 mM). All cell culture supplies were purchased from Gibco BRL, Paisley, UK. Cell lines were maintained at 37°C in a humidified, 5% CO_2 _environment. For UVA exposure, followed by RNA isolation and cDNA hybridization, 850000 B16-F1 melanoma cells were plated on the cell culture dish (area 55 cm^2^). Cells were grown for 48 hours, after which semi-confluent B16-F1 monolayers were formed and the melanoma cells were exposed to UVA dose of 8 J/cm^2^.

### Radiation source and irradiation procedure of B16-F1 melanoma cell

**Figure 4:**
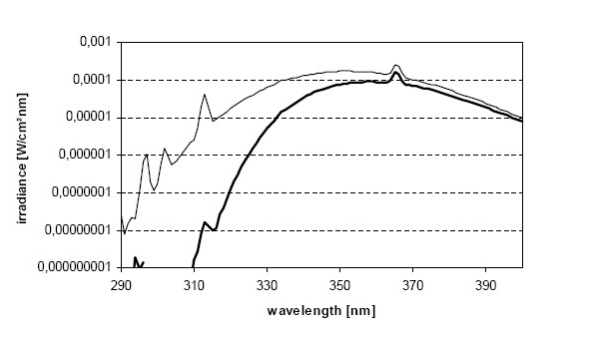
**UV spectrum irradiating B16-F1 mouse melanoma cells**. Spectral irradiance of a Philips HB 171/A face tanner filtered through a 5 mm glass plate (thin line) and filtered through a 5 mm glass plate and a culture dish cover (thick line).

An "Original Philips UVA" facial tanner model HB 171/A (Philips, Germany) was used as a radiation source. The irradiances of the UVA source were measured as described previously [14,28]. Briefly, the irradiances were determined with a temperature- and wavelength-controlled Optronic 742 double-holographic grating spectroradiometer having a Teflon diffuser as input optics at 1 nm intervals from 250 to 400 nm. The spectroradiometer was calibrated against a 1000 W halogen standard lamp traceable to the National Standards and Technology (USA) and the overall spectroradiometric uncertainty is estimated to be ± 8 %. The cells were irradiated in the plastic Petri dishes through a 5-mm thick glass filter to cut off residual UVB radiation, dish cover and culture medium and the UVA spectrum used was 310–400 nm, from which the UVA portion was 99.99% and UVB 0.01% (Figure 4). The UV irradiance that reached the melanoma cells was 3.7 mW/cm^2^. The culture medium was changed to room temperature medium prior to irradiation so that it does not exceed 37°C. The B16-F1 melanoma cells were exposed to single UVA dose of 8 J/cm^2^, which roughly corresponds to the UVA dose received approximately within 1 hour on a sunny summer day in Finland. All irradiations were performed at room temperature in a dark room on a black support to avoid effects of reflected radiation. The control cells were kept at room temperature in a dark room.

### RNA extraction

Cells were collected 4 hours after the end of UVA irradiation by brief trypsinization and washed twice with ice cold PBS. The total RNA was isolated from melanoma cells using Nucleospin^®^RNA II kits (Clontech Laboratories, Palo Alto, CA) and RNA concentrations were determined spectrophotometrically measuring the optical densities. The possible genomic DNA contamination was monitored using total RNA as template in a PCR reaction with primers for genomic β-actin [29]. The PCR products were run by agarose gel electrophoresis and only DNA free samples were used in Atlas™ Microarray procedure.

### The cDNA probe preparation and array hybridization

The poly A^+ ^RNA enrichment of 50 μg total RNA and ^32^P-labelled cDNA probe synthesis made by reverse transcription were performed according to Atlas™ Pure Total RNA Labelling System (Clontech Laboratories, Palo Alto, CA). Precisely the same amounts of ^32^P-labelled cDNA from control and UVA exposed melanoma cells were used as a probe in Atlas™ Mouse Cancer 1.2 cDNA expression arrays (Clontech Laboratories, Palo Alto, CA), containing 1176 tumor related genes immobilized on a nylon membrane. The whole list of the studied genes is found at the manufacturer's home page [30]. Hybridization and washing procedures were performed according to recommendations of the manufacturer (Clontech Laboratories, Palo Alto, CA). Briefly, the array membranes were pre-hybridized for 30 minutes at 68°C in ExpressHyb hybridization solution containing 0.1 mg/ml salmon testis DNA. The ^32^P-labelled cDNA was added to the hybridization solution and the array membrane was hybridized overnight at 68°C. On the next day array membranes were washed three times with 2 × SSC solution (NaCl 0.29 M, tri-sodium-citrate 0.029 M) supplemented with 1% SDS, and two times with 0.1 × SSC solution (NaCl 0.015 M, tri-sodium-citrate 1.5 × 10^-3^M) supplemented with 0.5% SDS. The x-ray film was exposed at -70°C simultaneously to the membrane hybridized with control probe and to the membrane hybridized with UVA exposed probe. The exposure time was exactly same with control and UVA exposed membrane within one experiment, but exposure times varied from 7 to 21 days between 5 experiments.

### cDNA array imaging and quantitation

Hybridization signals on the autoradiograms were scanned using GS-710 Calibrating Imaging Densitometer (Bio-Rad Laboratories, Hercules, CA) and the intensity of gene spots was analyzed by AtlasImage 2.0 Software (Clontech Laboratories, Palo Alto, CA). Analysis of the membranes was performed according to the manufacturer's instructions. The cut-off value to the reliable gene hybridization signal was set to 3000 OD and only those genes whose intensity was bigger than 3000 in either control or UVA exposed spot were included in the further examination. Thereafter, the intensity of gene was normalized against the average OD of nine housekeeping genes in the membrane. Ratio of two corresponding normalized gene spots between control and UVA exposed spots was calculated by dividing the intensity of normalized UVA exposed gene by the normalized intensity of control gene. To select the genes with altered expression level, the significance of up-regulation was set at ratio ≥ 1.7 and down-regulation at ratio ≤ 0.6. Subsequently, the existence of the spots on the film was verified by visual examination. The gene expression level was considered to be changed if the alteration was observed in three or more experiments out of five experiments. The student t-test was performed to calculate the statistical significance of the change.

### Western blot analysis of Cyclin G

B16-F1 melanoma cells were harvested, either immediately after the end of UVA exposure or at different time points, with versene (140 mM NaCl, 2.7 mM KCl, 8 mM Na_2_HPO_4_, 0.5 mM EDTA). Melanoma cells were washed twice with cold PBS and lysed with 2.5% sodium dodecylsulfate with 1% proteinase inhibitors (cat# P-8340, Sigma-Aldrich, St. Louis, MO, USA). The protein concentration was measured according to Lowry [31] using the Bio-Rad DC Protein Assay (Bio-Rad, Hercules, CA, USA). Samples containing 20 μg protein per lane were resolved using 7.5 % SDS-PAGE gel and blotted on PVDF membranes (Bio-Rad Mini-Protean^® ^3 cell apparatus, Hercules, CA, USA). Immunoblots were developed according to ECL Advanced kit (Amersham Biosciences, UK). Briefly, membranes were incubated at room temperature for one hour in a 2% blocking agent diluted in PBS with 0.5 % Tween and followed by incubation in the 1^st ^anti-cyclin G antibody (Neomarkers, Fremont, CA; diluted 1: 2000 in the blocking solution) overnight on a rotary shaker at +4°C. Membranes were washed with PBS supplemented with 0.5% Tween for 3 × 10 minutes and incubated in a secondary antibody solution of horseradishperoxidase-conjucated mouse anti-mouse immunoglobulin G (DAKO, Denmark; diluted 1:35000 in blocking solution) for 1 h at room temperature on a rotary shaker. Thereafter, immunoblots were washed for 3 × 10 minutes with PBS with 0.5% Tween and the signal was detected using chemiluminiscence (Amersham Biosciences, UK). Autoradiograms were scanned by GS-710 Calibrating Imaging Densitometer (Bio-Rad Laboratories, Hercules, CA) and the intensity of protein bands was analyzed by Phoretix 1D v2003.01 from three different set of experiments. The student t-test was performed to calculate the statistical significance of the change.

### Cell cycle analysis

The cell cycle was studied by flow cytometry examining the DNA content of B16-F1 cell by propidium iodide staining as described previously [32]. B16-F1 melanoma cells were collected with versene, either immediately after the end of UVA exposure or at different time-points thereafter, cells were washed twice with cold PBS, and fixed in methanol. After fixation, melanoma cells were washed twice with cold PBS followed by incubation in an RNase solution in PBS (100 units/ml) for 30 minutes at 37°C. Melanoma cells were incubated in propidium iodide solution in PBS (10 μg/ml) overnight at +4°C. Fluorescence was measured using FACScan flow cytometry (Becton Dickinson, USA) and analyzed with ModFitLT V3.1(PMac) cell cycle analysis program (Becton Dickinson, USA). The student t-test was performed to calculate the statistical significance of the change.

## Competing interests

The author(s) declare that they have no competing interests.

## Authors' contributions

RP and DL designed the study. RP carried out the experiments and prepared the manuscript. DL revised the final version of the manuscript. All authors read and approved the manuscript.
